# Comparison of cake compositions, pepsin digestibility and amino acids concentration of proteins isolated from black mustard and yellow mustard cakes

**DOI:** 10.1186/s13568-015-0110-y

**Published:** 2015-04-07

**Authors:** Ashish Kumar Sarker, Dipti Saha, Hasina Begum, Asaduz Zaman, Md Mashiar Rahman

**Affiliations:** Plant Protein Research Section, Institute of Food Science and Technology (IFST), Bangladesh Council of Scientific and Industrial Research( BCSIR), Dr. Kudrat-i-Khuda Road, Dhanmondhi, Dhaka, 1205 Bangladesh; Department of Applied Chemistry & Chemical Engineering, Dhaka University, Dhaka, 1000 Bangladesh; Enzymlogy Research Section, IFST, BCSIR, Dhaka, 1205 Bangladesh

**Keywords:** Mustard cake, Protein isolation, Pepsin digestibility, Amino acids analysis

## Abstract

As a byproduct of oil production, black and yellow mustard cakes protein are considered as potential source of plant protein for feed applications to poultry, fish and swine industries. The protein contents in black and yellow mustard cakes were 38.17% and 28.80% and their pepsin digestibility was 80.33% and 77.43%, respectively. The proteins were extracted at different pH and maximum proteins (89.13% of 38.17% and 87.76% of 28.80% respectively) isolated from black and yellow mustard cakes at pH 12. The purity of isolated proteins of black and yellow mustard cakes was 89.83% and 91.12% respectively and their pepsin digestibility was 89.67% and 90.17% respectively which assigned the absence of antinutritional compounds. It was found that essential amino acids isoleucine, lysine, methionine, threonine and tryptophan and non essential amino acids arginine and tyrosine were present in greater concentration in black mustard cake protein whereas other amino acids were higher in yellow mustard cake protein.

## Introduction

Oil cakes/oil meals are by-products obtained after oil extraction from the seeds. Oil cakes are of two types, edible and non-edible. Edible oil cakes have a high nutritional value; especially have protein contents ranging from 15% to 50% (Ramachandran et al. 2007). Black Mustard (*Brassica nigra*) and yellow mustard (*Sinapis alba*) are the third important oilseed crops in the world after soybean (*Glycine max*) and palm (*Elaeis guineensis* Jacq.) oil seed. These seeds are produced in Bangladesh in a large extent. They contain relatively high amount of protein with small amounts of anti nutritional compounds (Clandinin and Heard, 1968). Deoiled groundnut cake is commonly used as poultry feed ingredient. Though high in protein, groundnut cake is a poor source of essential amino acids like lysine and methionine; but commonly infested with *Aspergillus* sp., which will produce aflatoxins under favourable conditions (Adebesin et al. 2004). Soybean is a major protein source for humans and other animals. The protein composition of soybean seed is not ideal for human and animal nutrition because of the poor content of sulfur containing amino acids (Fukushima, 1991; Sievwright and Shipe, 1986) and presence of large amount of phytic acid. In this circumstance, deoiled mustard cakes appear to be a potential source of protein replacing ground nut and soybean cakes in fish and poultry rations.

Different methods have been examined for the production of protein isolate (>90% protein) and concentrates (>65% protein) from these cakes. Prapakornwiriya and Diosady used yellow mustard flour to successfully develop a microfiltration based process for production of protein concentrate (Prapakornwiriya and Diosady, 2004). Marnoch and Diosady used oriental mustard seed (*Brassica juncea* L.) to develop a membrane-based process that produced three products: a precipitated protein isolate (PPI), a soluble protein isolate (SPI) and a meal residue (Marnoch and Diosady, 2006). The protein isolates were high in protein and free of anti-nutritional compounds. High levels of protein, suitable amount of essential amino acids, minerals and the behavior ability of these nutrients have given mustard seed cakes prime importance as a quality protein source. Unfortunately, cakes or seeds contain some compounds like glucosinolates and their breakdown products, phenolics and phytates which hinder bioavailability of amino acids and minerals (Dijkstra et al. 2003; Naczk et al. 1992)_._ These compounds are responsible for the dark color and astringent flavor and they must be removed. Before incorporating deoiled mustard cakes in poultry ration, they should be analyzed for their proximate compositions, isolation of proteins, and pepsin digestibility of proteins and amino acid profiles of isolated proteins to know their nutritive value.

The objectives of the present work were to determine nutritional composition of cakes, isolation of proteins from cakes, pepsin digestibility of isolated proteins and cakes and analysis of amino acids composition of isolated proteins.

## Materials and methods

### Materials

Sodium hydroxide (NaOH), sulfuric acid (H_2_SO_4_), copper sulfate (CuSO_4._5H_2_O), potassium sulfate (K_2_SO_4_), hydrochloric acid (HCl), pepsin, petroleum ether, and ethanol were from BDH and used without further purification. Phosphate buffers (pH 8, 9, 10, 11, 12, 13 and 14) were prepared by mixing proper amount of 0.1 M disodium hydrogen phosphate and 0.1 M sodium hydroxide.

### Methods

#### Testing samples preparation

Black and yellow mustard cakes were used for this study and obtained from a local oil mills. Prior to use, the cakes were ground and defatted with petroleum ether (40-60°C), using a Soxhlet apparatus, for 16 h, and then dried overnight in an oven at 80°C. The moisture, minerals, crude fibre and fat of fat were determined by the standard AOAC method 950.46, AOAC method 920.153, AOAC method 985.29 and AOAC method 960.39 respectively (AOAC, 2005). Crude protein was determined by the micro-Kjeldahl method and reported as%N × 6.25 (AACC, 2000).

#### Removal of allylisothiocyanate

The allylisothiocyanates were removed by the modified method of Singh (Singh, 1988). 10 g defatted sample was grinded and passed through a No 20 sieve. 150 ml 5% ethanol was added in 6 g powdered sample in 300 ml Erlenmeyer flask, stopper tightly and magnetic stirred for 90 m at 37°C. After extraction, the cakes were filtered with mild vacuum and dried at 80°C for 8 h. The raw mustard cakes were also dried same temperature to compare the pepsin digestibility with allylisothiocyanate free cakes.

#### Determination of allylisothiocyanate

The quantity of allylisothiocyanate was determined by titrimetric method. Exactly 5 g raw mustard cakes were mixed with 12.5 ml absolute ethanol and 237.5 ml distilled water into a 500 ml distillation flask. The mixture was distilled with steam and 150 ml distillate was collected in the 25 ml 0.1 N silver nitrate and 10 ml 10% ammonium hydroxide solution. The distillate mixture was boiled for 1 h under air reflux in water bath, cooled, volume made upto 250 ml and then filtered. 100 ml filtrate was titrated with standard ammonium thiocyanate solution in acidic condition using few drops of ferric ammonium sulfate indicator. A blank titration was also done and calculated the amount of allylisothiocyanate.

#### Protein extraction

The extraction of protein was carried out according to the method of Marnoch and Diosady with small modification (Marnoch and Diosady, 2006). The protein extractability was determined by contacting 20 g of ground defatted mustard cakes with aqueous NaOH solution at a solvent to cakes ratio of 18 in a preset pH, ranging from 8 to 14. The pH was adjusted using phosphate buffer. The extract and solids were separated by centrifugation at 12000 rpm. The liquid was decanted and vacuum filtered through Whatman 41 paper to a receiving flask. The solids were washed twice with distilled water and each time decanted through the filter paper into the same receiving flask. The extractability was measured as the mass ratio of the recovered protein in the collected extract solution compared with that in the 20 g of starting material.

Protein was precipitated from extract solution by adding 1 M HCl solution. The pH of extracted protein solution was kept constant at 5 and allowed to stand for overnight at 5°C for precipitation. The precipitated protein isolate (PPI) was separated by centrifugation (10,000 rpm), for 15 min in a centrifuge machine (Kokusan, H2000 series). The PPI was then washed with water, dried by freeze drying and stored at 5°C for further analysis.

#### Protein digestibility

The in vitro protein digestibility of raw defatted cakes, allylisothiocyanate free cakes and PPI was carried out according to the method of Mertz et al. with a minor modification (Mertz et al., 1984). 2.0 g sample was mixed with 490 ml distilled water and 1.5 g pepsin. Then 10 ml 25% HCl was added and the final solution was incubated for 24 h at 37°C in incubator. After this treatment, further 6 h incubation at 37°C was done with additional 10 ml 25% HCl. After incubation the reaction was stopped by addition of 15 ml of 10% trichloroacetic acid (TCA). The mixture was filtered and washed with distilled water. The residue was collected and estimated the nondigest nitrogen by micro-Kjeldahl method.

#### Amino acids analysis

Amino acid composition of protein isolates was determined by using an amino acid analyzer (Shimadzu, Japan) and only fourteen amino acids were determined due to the limitation of the instrument. 0.5 g isolated protein was pasted with 50 ml 6 N HCl by mortar pestle, filter and then the filtrate was hydrolyzed 22–24 h in a hydrolysis tube. After hydrolyzing, HCl was removed from the filtrate by evaporating and three times re-evaporating with water in water bath. After evaporation, the solution was volume to 25 ml in volumetric flask by 0.1 N HCl. The stock solution was used for amino acids analysis using Shimadzu Amino Acid Analyzer.

#### Statistical analysis

Three replicates were carried out in each experiment. All data were analyzed by SPSS software, version 15 using one-way ANOVA analysis. The level of statistical significance was set at 5% (p < 0.05).

## Results

### Proximate chemical analysis

Proximate compositions of black mustard cake and yellow mustard cake are presented in Table 1. The black mustard cake contains higher crude protein of 38.17% than that of yellow mustard cake protein of 28.80% hence appeared to be a moderately good source of protein. On the other hand, ether extract content was high as 15.67% for yellow mustard cake than 8.70% for black mustard cake. Crude fibers content were also so high for both types of cakes. Fiber and oil content can be used to approximate energy values of the cakes, since utilizable energy content decreases as fiber content increases, but increases as the oil content increases. Thus, the cakes are a good source of energy, since they have high oil content however; the energy content is limited by the high fiber content. Total minerals content of black mustard cake and yellow mustard cake were found to be 7.10% and 5.90% among which contribution of acid insoluble ash were only 1.93% and 1.23% indicating that it is a good source of minerals. Allylisothiocyanate content in cakes is 0.086% and 0.077% respectively. It can bind with protein and decreased the digestibility of protein. After removal of this antinutritional compound the digestibility of protein increased.

**Table 1:** **Proximate composition of black mustard cake and yellow mustard cake**

**Materials**	**Moisture, %**	**Minerals, %**	**Acid insoluble ash, %**	**Oil, %**	**Crude fiber, %**	**Crude protein, %**	**Allylisothiocyanate %**
Black mustard cake	9.20 ± 0.5	7.10 ± 0.3	1.93 ± 0.4	8.70 ± 0.8	12.17 ± 1.3	38.17 ± 1.0	0.086 ± 0.009
Yellow mustard cake	9.73 ± 0.6	5.90 ± 0.3	1.23 ± 0.3	15.67 ± 0.6	14.80 ± 0.2	28.80 ± 0.7	0.077 ± 0.003

All data are the average of three replicate independent experiments and the standard deviation was calculated using one-way ANOVA.

### Protein extraction

**Figure 1:**
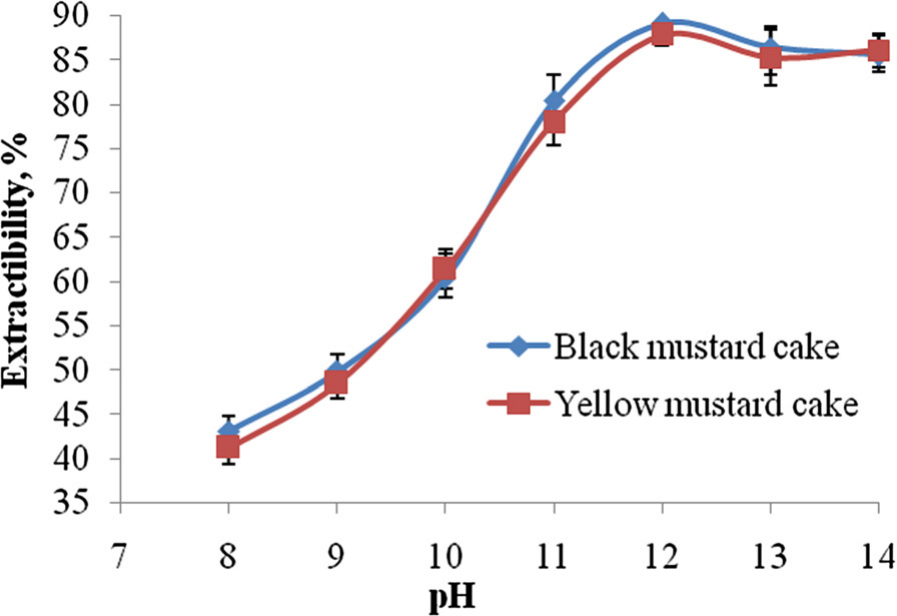
**Protein extraction from defatted ground mustard cakes by aqueous sodium hydroxide solutions as a function of pH (All data are the average of three replicate independent experiments and the standard deviation was calculated using one-way ANOVA).**

Protein extraction is normally governed by the pH values which influence the ratio of free to neutralized charges. The data of the study indicates that protein solubility was gradually enhanced with the increase in pH values from 8. However, maximum solubility of mustard cakes protein was increased rapidly from 38.17% to 89.13% at pH 12 for black mustard cake and from 28.80% to 87.76% at pH 12 for yellow mustard cake. The effect of pH on protein extractability is presented in Figure 1.

### Protein digestibility

The pepsin digestibility of three forms of protein: protein in raw mustard cakes, protein free from allylisothiocyanate and precipitated protein isolate (PPI) of black and yellow mustard cakes are shown in Table 2. In both mustard cakes and anti nutritional compounds free mustard cakes there were significant differences in protein digestibility. Protein concentrations in the raw black and yellow cakes were 38.17% and 28.80% respectively. In vitro protein digestibility values were 80.33% for black mustard cake and 77.43% for yellow mustard cake; whereas the antinutritional compounds free type mustard cakes had digestibility values of 89.67% for black mustard cake and 90.17% for yellow mustard cake. Except for two entries, the isolated protein had higher digestibility values 96.67% for black mustard cake and 95.27% for yellow mustard cake. The protein digestibility was slightly changed after preparation of protein isolate than cakes without antinutritional compounds. From the Table 2 it is shown that the protein digestibility both yellow and black mustard cakes increased about 16% in case isolated protein than the crude protein in mustard cakes.

**Table 2:** **Pepsin digestibility of protein in black mustard and yellow mustard cakes**

**Parameters**	**Black mustard cake**	**Yellow mustard cake**
Pepsin Digestibility of protein before removal of antinutritional compounds,%	80.33 ± 2.08	77.43 ± 1.53
Pepsin digestibility of protein after removal of antinutritional compounds,%	89.67 ± 1.15	90.17 ± 2.51
Pepsin digestibility of isolated protein,%	96.67 ± 1.53	95.27 ± 2.08

All data are the average of three replicate independent experiments and the standard deviation was calculated using one-way ANOVA.

### Amino acids composition

Amino acid composition of protein isolates is an indicator of their nutritive value. The concentrations of essential amino acids in black mustard cake protein isolate and yellow mustard cake protein isolate differ from each other considerably (Table 3). The isoleucine (5.57%), lysine (4.55%), methionine (2.52%), threonine (19.17%) and tryptophan (1.96%) content in black mustard cake protein isolate have greater concentration than in yellow mustard cake protein isolate while leucine (1.12%), valine (1.74%) and histidine (0.90%) content in yellow mustard protein isolate have higher concentration than black mustard cake protein isolate. The non essential amino acids such as arginine (2.74%) and tyrosine (1.96%) in black mustard cake protein isolate and alanine (4.26%), aspartate (7.11%), glycine (5.55%) and serine (5.03%) in yellow mustard cake isolate are found in higher concentration.

**Table 3:** **The composition of amino acids in black and yellow mustard cake protein isolates**

**Essential amino acids**			**Other non-essential amino acids**		
**Amino acids**	**Precipitated protein isolate (PPI) from black mustard cake (%)**	**Precipitated protein isolate (PPI) from yellow mustard cake (%)**	**Amino acids**	**Precipitated protein isolate (PPI) from black mustard cake (%)**	**Precipitated protein isolate (PPI) from yellow mustard cake (%)**
Isoleucine	5.57	2.95	Alanine	3.56	4.26
Leucine	0.83	1.12	Arginine	2.74	2.28
Lysine	4.55	2.70	Aspartate	4.49	7.11
Methionine	2.52	1.50	Glycine	2.54	5.55
Threonine	19.17	14.31	Serine	2.95	5.03
Tryptophan	1.96	1.39	Tyrosine	1.96	1.39
Valine	1.20	1.74			
Histidine	0.43	0.90			

## Discussion

Mustard cakes is a potential source of protein for animals and it is the first study to compare the nutritional, isolation of protein, pepsin digestibility of protein and amino acids pattern of black and yellow mustard cakes. As shown in Table 1, moisture content of black and yellow mustard cakes were 9.20 ± 0.5% and 9.73 ± 0.6%. The results of the moisture content were slightly higher than the moisture content 8.3 ± 0.2% reported in literature. (Al Mahmud et al. 2012; Marnoch and Diosady, 2006)*.* Excess water used in the moisturizing of mustard seeds during oil extraction is responsible for this reason. The Crude protein content of mustard cakes obtained were 38.17% and 28.80% which were lower (45.0% and 34.0%) than those reported by many other authors (Marnoch and Diosady, 2006; Prapakornwiriya and Diosady, 2004). However, Chowdhury et al. and Kumar ent al. reported comparatively equal amount of crude protein in mustard cakes (Anil Kumar et al., 2002; Chowdhury et al. 2010). The variation observed could be because of the variety of mustard raised and the differences in sampling adopted, influence of season of harvest etc. Mean ether extract content of black and yellow mustard cakes were also low compared to many other authors (Latif et al., 2008; Ramachandran et al., 2007). Crude fiber content of mustard cakes were 12.17 ± 2.25% which was also found to vary among report of different authors (Latif et al., 2008; Sharma et al., 2012). Variations due to hulling procedure and time, variety etc., would have contributed to these differences. Ash content of mustard cakes were 7.10 ± 0.3% and 5.90 ± 0.3% respectively. These data are comparable (7.12 ± 0.12%) to the results reported in literature (Datta et al., 2013). Allyl isothiocyanates content of black and yellow mustard cakes were 0.086 ± 0.009% and 0.077 ± 0.003% which were comparable to the report of Sharma et al. (Sharma et al., 2012).

Various techniques for extraction and precipitation of protein isolates have been published. The alkaline extraction and acidic precipitation were chosen to produce PPI because this technique ensured maximum protein recovery and minimum protein denaturation. When pH value is above 5, the solubility of protein will be increased and hence the extraction of protein from mustard cakes gradually increased with increasing pH values from 9. The effect of pH demonstrated in this experiment is in agreement with the pH values reported in the literature (Lindeboom and Wanasundara, 2007; Marnoch and Diosady, 2006). The maximum extractability of proteins 89.13% and 87.76% was observed at pH 12.0. These extractions of proteins were also comparable to the reports of Marnoch and Diosady (Marnoch and Diosady, 2006). Buffer composition and filtration method had no effect for separation of protein from mustard cakes whereas pH is the only factor for separation of protein from cakes.

Pepsin digestibility of protein is an important chemical property of proteins, as it determines their nutritional value. The digestibility increased after separation of allylisothiocyanates from cakes. The pepsin digestibility of PPI was highest compared with the protein of allylisothiocyanates free cakes and protein of raw cakes. Therefore, the antinutritional compounds have negative effect on protein pepsin digestibility (Aparicio-Saguilán et al., 2015; Sun et al., 2012). The high fibre content in raw cakes and allylisothiocyanate free cakes may also affect the pepsin digestibility of protein. From the Table 2 it is shown that the protein digestibility both yellow and black mustard cakes increased about 15% in case of isolated protein than the crude protein in mustard cakes. This results assigned that PPI was free of anti nutritional compounds and there was no scope for lysinoalanine formation during extraction Some examples of naturally occurring antinutritional factors include glucosinolates in mustard and rapeseed protein products which would adversely affect nutrient utilization and may contribute to growth depression in animals (Fenwick et al., 1982). All of these tend to make the amino acids less available and, in general, the protein less digestible (Talati et al., 2004). Amino acid composition of PPI from black and yellow mustard cakes indicated that it is a good source of lysine and methionine. However, essential amino acids such as isoleucine, lysine, methionine, threonine and tryptophan contents were found to be higher in black mustard protein than in yellow mustard protein. The decrease of the lysine amounts in the protein isolates is probably due to the interaction of the respective amino acid with other plant components during oil processing.

Our experimental results indicate that black mustard cake is more beneficial than yellow mustard cake for preparation of feeds. The use of this byproduct, as a part of a protein extraction process would increase the viability of the linked industrial processes.
